# Long Non-coding RNAs Influence Aging Process of Sciatic Nerves in SD Rats

**DOI:** 10.2174/1386207326666230907115800

**Published:** 2023-09-07

**Authors:** Rui Kuang, Yi Zhang, Guanggeng Wu, Zhaowei Zhu, Shuqia Xu, Xiangxia Liu, Yangbin Xu, Yunxiang Luo

**Affiliations:** 1 Department of Plastic Surgery, The First Affiliated Hospital of Sun Yat-Sen University, No. 58 Zhongshan Road 2, Guangzhou 510080, China;; 2 Department of Plastic Surgery, University of Tennessee Health Science Center, Memphis, TN, United States

**Keywords:** Long non-coding RNAs, peripheral nerves, aging, transcriptome, bioinformatics, DNA

## Abstract

**Objectives:**

To investigate the long non-coding RNAs (lncRNAs) changes in the sciatic nerve (SN) in Sprague Dawley (SD) rats during aging.

**Methods:**

Eighteen healthy SD rats were selected at the age of 1 month (1M) and 24 months (24M) and SNs were collected. High-throughput transcriptome sequencing and bioinformatics analysis were performed. Protein-protein interaction (PPI) networks and competing endogenous RNA (ceRNA) networks were established according to differentially expressed genes (DEGs).

**Results:**

As the length of lncRNAs increased, its proportion to the total number of lncRNAs decreased. A total of 4079 DElncRNAs were identified in Con *vs.* 24M. GO analysis was primarily clustered in nerve and lipid metabolism, extracellular matrix, and vascularization-related fields. There were 17 nodes in the PPI network of the target genes of up-regulating genes including Itgb2, Lox, Col11a1, Wnt5a, Kras, *etc*. Using quantitative RT-PCR, microarray sequencing accuracy was validated. There were 169 nodes constructing the PPI network of down-regulated target genes, mainly including Col1a1, Hmgcs1, Hmgcr. CeRNA interaction networks were constructed.

**Conclusion:**

Lipid metabolism, angiogenesis, and ECM fields might play an important role in the senescence process in SNs. Col3a1, Serpinh1, Hmgcr, and Fdps could be candidates for nerve aging research.

## 1 INTRODUCTION

Aging has always been an unavoidable topic in society nowadays. Statistics show that life expectancy in many developed countries in the world is more than 80 years [1]. Due to the increase in life expectancy, people also pay growing attention to neurodegenerative diseases related to nerve aging. Like other tissues of the body, peripheral nerves (PNs) inevitably age as an individual gets older. The process of nerve aging is mainly reflected in the progress of the pathological process and the decreased normal physiological function of cells [2]. Specifically, this series of changes includes altered metabolic patterns, reduced adaptation to stress, the accumulation of damaged proteins, lipids, and deoxyribonucleic acid (DNA), and pathobiological and traumatic aspects like accelerated cellular senescence and neurodegeneration [3, 4].

In molecular biology, one of the main principles is to transcribe messenger ribonucleic acid (mRNA) from DNA and then translate it into protein. This “central dogma” was proposed more than five decades ago. Therefore, the monitoring mechanisms that describe transcription and translation have been the fundamental laws of biology, with profound implications for health and disease [5]. Recent advances in the next-generation RNA sequencing (NGS) of the human genome show that only one-fifth of total transcription occurs in protein-coding genes. This suggests that quantities of non-coding RNA (ncRNA) species exist in the mammalian transcriptome. Those high-resolution maps of the transcriptome uncover long non-coding RNAs (lncRNAs) involved in regulating many aspects of gene transcription, including basic transcripts, microRNA regulation, and epigenetic phenomena [6].

It was previously hypothesized that the amount of myelin-associated protein mRNA synthesis gradually decreased with age. However, the resulting protein was gradually deposited in nerve fibers in a “tree-ring” manner, and myelin was formed with the increase in thickness [7, 8]. To further figure out whether the role of lncRNAs in the nerves of rats at different ages has similar changes to mRNA, the sciatic nerves (SNs) of Sprague Dawley (SD) rats at different life stages (physiological aging process at 1 and 24 months) were used in this study.

The purpose was to evaluate the potential contribution of gene and protein changes, which may affect the aging and regeneration of PNs. The differentially expressed genes (DEGs) in SNs were analyzed by sequencing and bioinformatics analyses, including the expression of differentially expressed mRNAs (DEmRNAs) and lncRNAs (DElncRNAs). Gene ontology (GO) analysis was conducted and a competing endogenous RNA (ceRNA) network was constructed, to identify the key biological processes (BP) and signal pathways associated with the regulation of lncRNAs during aging. This study hoped to seek age-related changes in the PN system (PNS) and tried to provide a theoretical prospective basis by observing the expression and function of important genes during the aging process.

## 2 MATERIALS AND METHODS

### 2.1 Animals

Twelve healthy SD rats were selected as the study models at the age of 1 month (1M) and 24 months (24M), with 6 animals in each group. All animals were bought from the Experimental Animal Research Center of Sun Yat-sen University, China. The study was approved by the Institutional Animal Care and Use Committee (IACUC), Sun Yat-Sen University, with the approval number SYSU-IACUC-2022-001797. All experimental operations were in line with the rules and regulations set by the IACUC. The harm and pain caused to animals during the experiment was minimized.

### 2.2 Specimen Collection

Surgical procedures of all rats were conducted as previously reported [8, 9]. All rats were anesthetized with ketamine hydrochloride (100 mg/kg) and 15 mm SNs were exposed under aseptic conditions bilaterally. Three rats were randomly selected from each group for sequencing.

### 2.3 High-throughput Transcriptome Sequencing

High-throughput transcriptome sequencing was performed by Shanghai Biotechnology Corporation (Shanghai, China). The detailed procedure was described as published before [9]. In brief, total RNA was extracted using RNeasy Micro Kit (Cat# 74004, Qiagen) following the manufacturer’s instructions and checked for an RIN number to inspect RNA integrity by an Agilent 2100 Bioanalyzer /Agilent 4200 TapeStation (Agilent Technologies, Santa Clara, CA, US). Qualified total RNA was further purified by RNAClean XP Kit (Cat A63987, Beckman Coulter, Inc. Kraemer Boulevard Brea, CA, USA) and RNase-Free DNase Set (Cat#79254, QIAGEN, GmBH, Germany). NanoDrop ND-2000 spectrophotometer/Qubit 2.0 and Agilent 2100 Bioanalyzer/Agilent 4200 TapeStation (Agilent Technologies, Santa Clara, CA, US) were applied for quality inspection, and only the qualified RNA could be used for subsequent sequencing experiments.

During transcriptome sequencing, the reads were converted into FPKM (fragments per kilobase of transcript per million mapped reads) to normalize the gene expression level. String tie (version: 1.3.0) was used to count the number of fragments for each gene after HisAT2 alignment, and then we used the TMM (trimmed mean of M values) method for normalization; finally, Perl script was used to calculate the FPKM value for each gene. Analysis of DEGs among samples was performed using the edgeR method.

### 2.4 qRT-PCR Verification

Quantitative analysis of the targeted mRNA expression of PNs in each age group (n = 3) was performed using RT-qPCR as previously described [8]. Using TRIzol reagent (Life Technologies, Carlsbad, CA, USA), total RNA (0.5 µg) was isolated from the collected SNs from each age group. Then, using the Revert Aid First Strand cDNA Synthesis Kit (Thermo, Massachusetts, USA), total RNA samples were reverse transcribed to cDNA. qPCR was performed using FastStart Universal SYBR Green Master Mix (Rox) (Roche, Basel, Switzerland) with specific primer pairs on a StepOne Plus Real-Time PCR system (Applied Biosystems, CA, USA). The primer pair sequences are listed in Supplementary Table **1**. All samples were run in triplicate. GAPDH was selected as the reference gene to normalize the target mRNA expression. *Via* routine agarose gel electrophoresis and melting curve analysis, the specificity of real-time PCR was confirmed. The 2^-ΔΔCt^ method was used to calculate relative gene expression.

### 2.5 Establishment of Protein-protein Interaction (PPI) Networks and ceRNA Networks

GO analysis (http://www.geneontology.org) was performed, with the standard that q-value ≤ 0.05 and a fold change≥2, to determine the roles of DElncRNAs. We used the online tool STRING (https://cn.string-db.org/) to map the PPI network of target genes of DElncRNAs. The specific procedures are the same as described in our previous report [8]. The sequences of all DElncRNA (FC >|2|, FDR < 0.05) were extracted according to the location information. The gene data of RT-PCR conform to a normal distribution and were presented as the mean ± SD. SPSS 27.0 software (SPSS, Chicago, IL, USA) was used to analyze the accuracy of NGS. Quantitative RT-PCR results were compared with sequencing results using Spearman's correlation coefficient method. **p < 0.05 was considered statistically significant. Predicting and comparing the targets of DElncRNAs and DEmiRNAs were achieved by the StarBase database, with Pearson correlation coefficient (PCC) > 0.95 as the criterion, to construct a lncRNA-miRNA-mRNA molecular interaction diagram.

## 3 RESULTS

After the NGS and bioinformatics analysis of SNs in rats aged 1 month and 24 months, the DElncRNAs and DEmRNAs of SNs in these groups were identified and used for subsequent research.

The raw data were uploaded on the Sequence Read Archive (SRA) data bank in the National Center for Biotechnology Information (NCBI): https://dataview.ncbi.nlm.nih.gov/object/PRJNA980627?reviewer=l1o1kivdqli642mfu0n73j3hsa.

### 3.1 Characteristic Analysis of LncRNAs

**Fig. (1):**
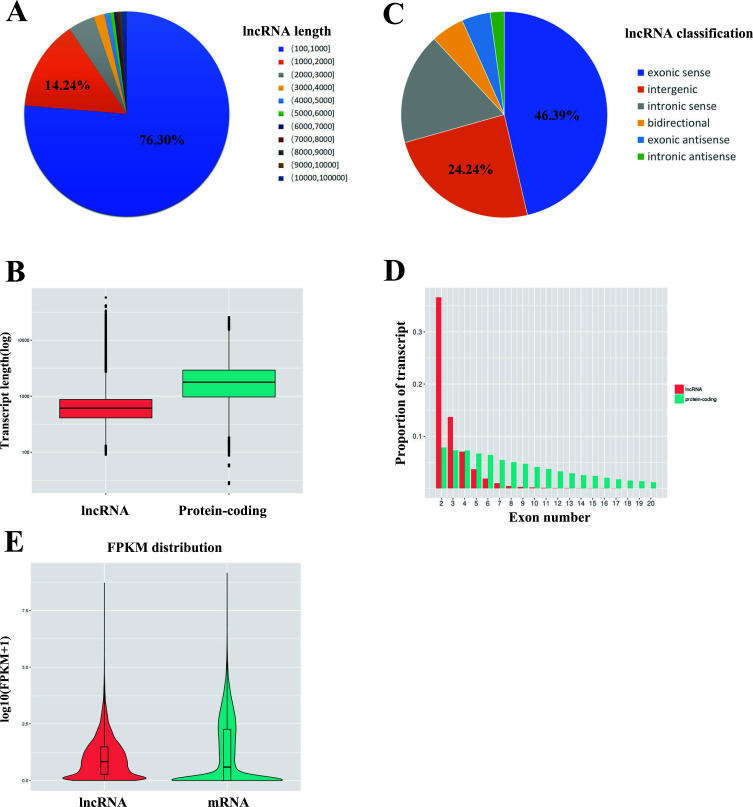
The features of lncRNAs derived from SN. (**A**) The length distribution of lncRNAs. (**B**) The average length of lncRNAs and mRNAs. (**C**) The classification of lncRNAs. (**D**) Exon number distribution per transcript of lncRNAs and mRNAs. (**E**) The FPKM of lncRNAs and mRNAs.

Firstly, the lncRNAs identified by sequencing were classified and counted according to their length. The results showed that the proportion of lncRNAs in the total number of lncRNAs decreased with the increase of their length (Fig. **1A**). The proportion of lncRNAs with a length of 100-2,000 nt was the highest, which reached 76.30%, and lncRNAs with a length of less than 4,000 accounted for the vast majority (>95%) (Fig. **1A**). The logarithm of lncRNAs and the length of protein-encoding genes was taken. The average length of lncRNAs was slightly less than 1,000, while the length of mRNAs was higher than that of lncRNAs (Fig. **1B**).

Meanwhile, the location of lncRNAs in the genome was classified as well. LncRNAs derived from the exonic sense region were found to occupy the highest proportion (46.39%), followed by intergenic lncRNAs (24.24%), whereas those derived from the intronic region accounted for the lowest proportion (2.18%) (Fig. **1C**). Then, the statistical analysis of lncRNAs and mRNAs based on the number of exons showed that lncRNAs with only one exon took up the largest proportion (34.82%), and over 95% of lncRNAs only contained less than five exons (Fig. **1D**). It is generally believed that the number of exons is positively correlated with the length of lncRNAs to a certain extent. In the meantime, the experimental results demonstrated that the number of exons of mRNAs was more evenly distributed compared with that of lncRNAs. In addition, 95% of mRNAs possessed at most 28 exons (Fig. **1D**). Finally, the average expression values of lncRNAs and mRNAs were analyzed, which indicated that lncRNAs had a slightly higher average expression level than mRNAs, and the distribution of expression values was more concentrated (Fig. **1E**).

### 3.2 Analysis and Sectionalization of DElncRNAs

The expression of lncRNAs aged between 1 (newborn, SNs) and 24 months (24M, senile SNs) was compared. In the current study, the specimen of one-month-old rats was set as the control group *(*Con), and the lncRNA expression patterns of Con *vs.* 24M were compared.

**Fig. (2):**
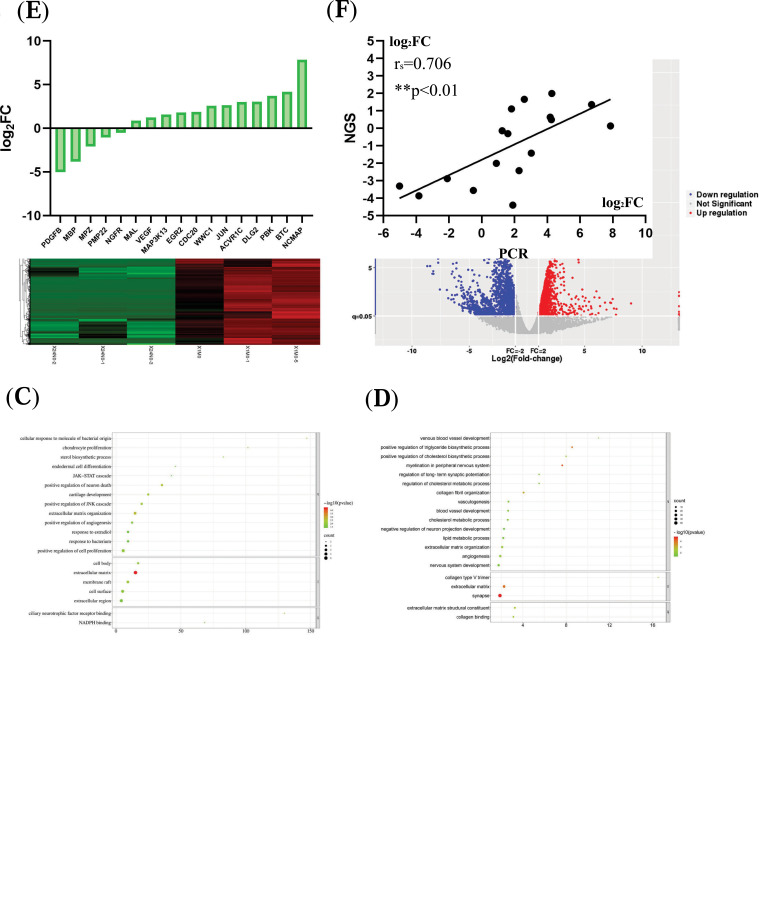
The DElncRNAs and DEmRNAs in different time points. (**A**) Heatmap of lncRNAs expression in Con and 24M group. (**B**) Volcano map of lncRNAs the Con *vs.* 24 M groups. (**C**) GO enrichment of up-regulated DElncRNAs in Con vs 24M groups. (**D**) GO enrichment of down-regulated DElncRNAs in Con vs 24M groups. (**E**) Quantitative RT-PCR (n = 3/group) and results of differentially expressed mRNAs in the sciatic nerve of 1M and 24M SD rats. To achieve baseline consistency, the quantitative RT-PCR fold change data were expressed as log_2_FC. FC: fold change; RT-PCR: real-time polymerase chain reaction. (**F**) Scatter map of Spearman correlation coefficient analyses between quantitative real-time polymerase chain reaction and sequencing data (Seq) of differentially expressed mRNAs (DEmRNAs) in the sciatic nerve of 1M and 24M SD rats. The correlation coefficient r_s_ was 0.706, and **p< 0.01 (n =3/group).

In terms of DElncRNAs, a total of 4,079 DElncRNAs were identified in Con *vs.* 24M, of which 368 were up-regulated and 3,711 were down-regulated (Fig. **2A**). Similar trends could also be found in volcano maps (Fig. **2B**).

### 3.3 GO/Kyoto Encyclopedia of Genes and Genomes Enrichment Analysis of DElncRNAs

The GO enrichment analysis of these target genes in each group was performed, and the screening criteria were set as *p*-value < 0.05. As shown in Fig. (**2C**), the target mRNAs of up-regulated DElncRNA in Con *vs.* 24M were mainly enriched in sterol biosynthetic process, Janus kinase-signal transducers and activators of transcription (JAK-STAT) cascade, the positive regulation of neuron death and JNK cascade, extracellular matrix (ECM) organization in BP (Fig. **2C** and Supplementary Table **2**). In cellular components (CCs), genes were mainly enriched in ECM (Fig. **2C**, Supplementary Table 2). When the molecular function (MF) was taken into consideration, genes were enriched in ciliary neurotrophic factor receptor binding (Fig. **2C**, Supplementary Table **2**). As presented in Fig. (**2D**), the GO enrichment analysis of target genes of down-regulated DElncRNAs in Con *vs.* 24M was conducted. When a comparison was made of Con *vs.* 24M, most of the down-regulated targets were enriched in the development of venous blood vessels, the positive regulation of triglyceride/cholesterol biosynthetic process, myelination in PNS and the regulation of long-term synaptic potentiation in BP subset (Fig. **2D**, Supplementary Table **3**). They were mainly located in collagen (Col) type V trimer, ECM and synapse in CC subset (Fig. **2D**, Supplementary Table **3**) and enriched in ECM structural constituent and Col binding in MF subset (Fig. **2D**, Supplementary Table **3**).

The Kyoto Encyclopedia of Genes and Genomes (KEGG) enrichment analysis of these target genes in each group was conducted, and the screening criteria were set as *p*-value < 0.05. It was found that the target mRNAs of DElncRNA in Con *vs.* 24M were mainly enriched in lipids and atherosclerosis, advanced glycation end products-receptor for advanced glycation end products (AGE-RAGE) signaling pathway in diabetic complications, and cortisol synthesis and secretion (Supplementary Tables **4** and **5**).

### 3.4 Quantitative Real-time Polymerase Chain Reaction Verification

Several genes (n = 17) were randomly selected from the sequencing of SD rats for quantitative real-time-polymerase chain reaction (qRT-PCR) quantitative analysis. The housekeeping gene glyceraldehyde-3-phosphate dehydrogenase (GAPDH) was used as an internal control to compare the sequencing results. A total of 17 groups were selected for qRT-PCR (Fig. **2E**) [8]. To evaluate the accuracy of NGS sequencing, Spearman correlation coefficient analysis was used to assess the correlation between RNA sequencing and qRT-PCR results. It was observed that the majority of these genes (n = 11) showed a consistent trend. The correlation coefficient r_s_ was 0.706, **p < 0.01, which indicated that the NGS results were positively correlated with qRT-PCR results (Fig. **2F**).

### 3.5 Analysis of Protein-protein Interaction Networks

**Fig. (3):**
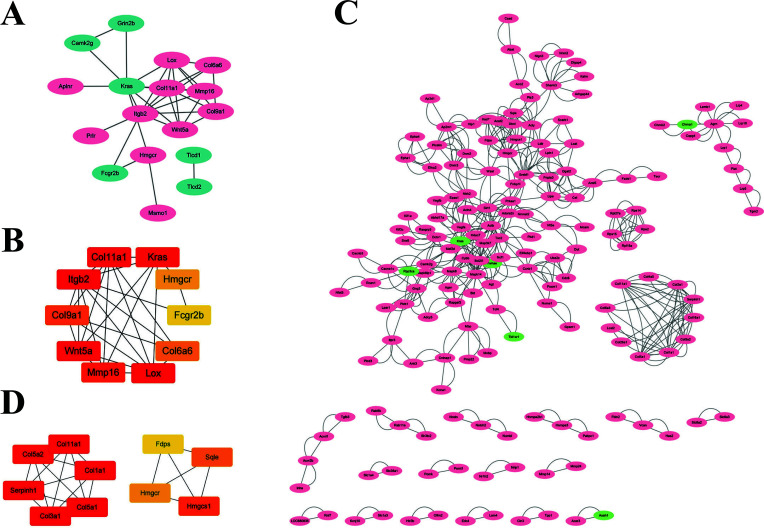
PPI networks: (**A**) The PPI network of the target genes of up-regulated DElncRNAs. and (**B**) Top 10 nodes in the PPI network of the target genes of up-regulated DElncRNAs. (**C**) The PPI network of the target genes of down-regulated DElncRNAs. and (**D**) Top 10 nodes in the PPI network of the target genes of down-regulated DElncRNAs. (Pale green nodes represent the up-regulated target gene, while pink nodes represent the down-regulated target gene).

Further, STRING was utilized to construct the PPI networks of DElncRNA target genes in Con *vs.* 24M. The networks were constructed according to the variation trend of genes separately. The PPI network of the target genes of up-regulating genes contained 17 nodes (Fig. **3A**), including integrin beta 2 (Itgb2), Lox, Col type XI, alpha 1 (Col11a1), wingless-type MMTV integration site family member 5a (Wnt5a), Kirsten rat sarcoma viral oncogene homolog (Kras), *etc*. The top 10 hub nodes in the network are shown in Fig. (**3B**). Additionally, 169 nodes constructed the PPI network of down-regulated target genes, mainly including Col1a1, human 3-hydroxy-3-methylglutaryl-coenzyme A synthase 1 (HMGCS1), 3-hydroxy-3-methylglutaryl coenzyme A reductase (HMGCR), *etc*. (Fig. **3C**). The top 10 hub nodes in the network are shown in Fig. (**3D**).

### 3.6 Construction of ceRNA Networks Based on LncRNAs

**Fig. (4):**
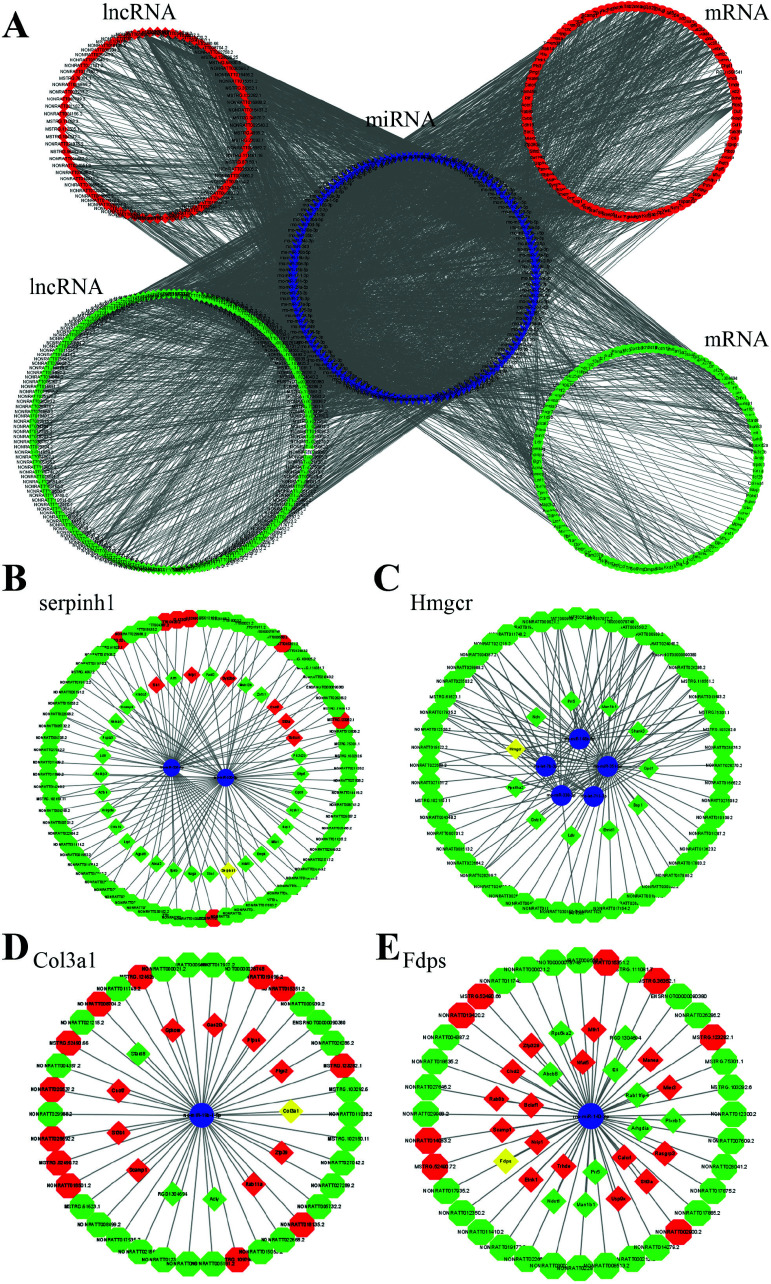
Construction of nerve senescence-related ceRNA network based on differentially expressed mRNA. (**A**). Nerve senescence-related ceRNA network. The central gene of each figure is (**B**) serpinh1, (**C**) Hmgcr, (**D**) Col3a1, (**E**) Fdps. (Red indicates up-regulation of expression, green indicates down-regulation of expression, hexagon indicates DElncRNA, rhombus indicates DEmRNA, and circle indicates microRNAs.).

Considering the importance of down-regulating lncRNAs during aging, the lncRNA-miRNA-mRNA-ceRNA interaction network was constructed *via* the bioinformatic method (Fig. **4A**). According to the PPI results, four molecules were chosen to establish target mRNA-related ceRNA networks, including Col type III alpha 1 (Col3a1), serpin peptidase inhibitor, clade H (heat shock protein 47), member 1 (Serpinh1), HMGCR and farnesyl diphosphate synthase (FDPS) (Fig. **4B-E**).

## 4 DISCUSSION

The senescence process in PNS involves some molecular and cellular processes, including but not limited to the following aspects: lipid metabolism, myelinated Schwann cells (SCs), blood vessels, axons, and ECM (Fig. **2C** and **D**). To be specific, the myelin sheath is one of the most important structures that support the function of nerves in PNS (Fig. **2D** and Supplementary Table **3**) [10, 11].

SCs are also important participants in neural aging. Clear evidence shows that the peripheral regeneration of aging PNS is inhibited by aged SCs [2, 12]. The close interaction between blood vessels and the nervous system involves the shared molecular mechanism of coordinating development and regeneration [13]. Extracellular environmental molecules constantly construct and regulate the formation and changes of nerves and blood vessels [14]. SCs, the main glial cells in PNS, coordinate most of the pathophysiological processes that develop in nerves [15, 16]. Success in nerve regeneration concerning peripheral neuropathy is attributed to changes in SC responses.

Such responses include their ability to secrete inflammatory mediators, remove cells and myelin fragments, and produce a wide range of regenerative factors that make the modified SCs a great coordinator of nerve repair [7, 17, 18], The results of this study confirmed the downregulation of related gene expression about vessels, lipids, and myelination (Fig. **2D**, Supplementary Table **3**). It has been shown that senescence affects the regeneration potential of PNS mainly by altering the plasticity of SCs, which further affects connective tissues and macrophages [2, 19].

### 4.1 Role of lncRNAs in Research on PNs

Mounting evidence shows that lncRNAs have complex functions, including activating or decreasing the expression of specific genes, especially adjacent protein-coding genes. Moreover, they are related to the pathogenesis of some nervous system diseases [20]. In brief, the molecular interactions of lncRNAs mainly include: transcribing the upstream promoter region of the protein-coding gene, Inhibiting RNA polymerase II, and forming complementary double chains with the transcript of the protein-coding gene, *etc.* [21, 22].

An increasing body of evidence shows that ncRNAs get involved in the physiological processes of central and PNSs [23-25]. A large number of studies have emphasized that it is important to better understand the biology of lncRNA regulation and function [6].

LncRNAs are widely involved in nerve growth and development. At present, a great amount of research focuses on the fields of neuropathic pain [26, 27] or regeneration after the injury of PNs [23, 28], *etc*. Currently, very few studies about lncRNAs are related to the natural aging of nerves with age. This article filled up the scientific field of changes in lncRNAs in aging nerves to some degree. It was noticed that the senescence of SNs is more manifested in nerve and lipid metabolism, ECM, and vascular-related fields after the analysis of transcriptional sequencing data from aging rats.

### 4.2 Nerve and Lipid Metabolism

Scores of studies have shown that neural aging is associated with changes in axons or synapses [7]. In this research, the target genes of up-regulated DElncRNAs tended to be located in synapses in the 24M group, and BP analysis was figured out in the regulation of long-term synaptic potentiation, synapse assembly, *etc* (Fig. **2C**, Supplementary Table **2**).

However, the senescence of PNs involves changes in transcriptional profiles, especially in lipid metabolism and immune responses (Fig. **2C** and Supplementary Table **2**) [4]. Previous studies speculated that the synthesis amount of myelin-associated protein mRNAs decreased with age [7, 8]. Another research has shown that lipids, including cholesterol, are important in regulating cell function, and cholesterol metabolism affects nerve regeneration during aging [29]. Researchers also observed demyelination and significant structural changes in the myelin sheath in old rodents, monkeys, and human brains [25]. Because of these results, changes in myelin sheath were recognized as an indicator of aging in medicine [30].

In the current study, an enrichment pathway in myelination in PNS was found in aging animals, which suggested that changes in myelin-related components might result from the regulation of lncRNAs [8]. When PPI networks were evaluated, it was found that several lipid-related hub genes in Con *vs.* 24M, like squalene monooxygenase (SQLE) and HMGCR, could further construct related ceRNA networks (Fig. **3**). These results strongly implied that lipid metabolism has a close relationship with nerve function.

### 4.3 ECM

It has been reported that the lack of axonal regeneration in old animals was ascribed to the dominant external inhibition of neuronal growth by the old microenvironment [19]. Plenty of nerve-related cells play an intercellular role in ECM. With the advent of the senescence of PNS, a significant decrease occurred in the Col fiber content and nerve fiber quantity in the nerve fasciculus [31]. Biochemical changes like protease-activated neuropeptide secretion in local ECM have an indirect influence on the plasticity and hardness of extracellular components [32]. In this research, a large number of down-regulated pathways about ECM were found in Con *vs.* 24M, including Col type V trimer, the negative regulation of cell-substrate adhesion, ECM organization and ECM (Fig. **2D**, Supplementary Table **3**). It was also noted that myriads of Col targets appeared in PPI networks, such as Col1a1, Col11a1, Col5a1, Serpinh1 and Col3a1. They could also be found in ceRNA networks (Fig. **3**).

As a most important component of ECM [33], Col is undoubtedly the most frequently used natural protein in the nervous system [34, 35]. Besides, it is widely used as a biological scaffold for nerve regeneration [36-38]. Col is shown to be able to help nerve regeneration with/without growth factors [39]. It can provide not only significant biochemical and mechanical support for nerve growth after injury [40] but also molecular adhesion and cell binding sites for supporting cells [37, 41-43]. As a result, axonal regrowth driven by the ECM-SC-neuron axis in the biomechanical microenvironment has been well accepted [44].

### 4.4 Vascular-related Fields

Neurovascular tissues form congruent patterns, whose three-dimensional structure establishes vital interactions between neural and vascular tissues [45]. In the process of nerve regeneration, angiogenesis plays a significant role in assisting nerve repair [46, 47]. Furthermore, angiogenesis is critical for the exercise-mediated enhancement of axon regeneration [48], which is opposite to unaccelerated aging [49]. This is consistent with the result of this study that numerous vascular-related pathways were down-regulated in Con *vs.* 24M groups, including venous blood vessel development, angiogenesis, blood vessel development, *etc*. (Fig. **2D**, Supplementary Table **3**). Materials that promote angiogenesis are more likely to promote neurogenesis [49, 50].

Apart from the above-mentioned major domains, other pathways and genes were also found to be involved in neural aging. In the field of glucose metabolism, for example, the glucagon signaling pathway was found to be up-regulated in KEGG-enriched pathways and involved in the neural aging process (Supplementary Tables **4** and **5**). Numerous studies have shown that neuropathy and aging are linked with glucose regulation and glucagon [51]. It has been demonstrated that the AGE-RAGE signaling pathway plays an important role in nerve repair, whose down-regulation in this study was in line with previous studies (Supplementary Tables **4** and **5**) [52]. Thus, it could be speculated that glucose metabolism has a profound effect on nerve growth and aging.

The past 25 years have witnessed an explosion in mining the genetic basis of nervous system diseases [53]. In the current study, many RNA-related therapeutic targets that may contribute to possible future therapeutic strategies were excavated. The senescence of nerves also involves a variety of changes including interactions between internal and external environments in PNS in addition to the aspects discussed above. This not only affects the clearance of myelin, but also changes the chemotaxis of macrophages, and even influences the process of immune regulation [19, 54, 55]. Both lncRNAs and mRNAs can affect the transcription and translation of nerve cells, which is of great significance for the study of PN aging and provides effective strategic significance for neuroscience to delay and treat PN degenerative diseases in the future.

## CONCLUSION

The changes of lncRNAs and mRNAs related to lipid metabolism, angiogenesis, and ECM are involved in the senescence of PNs. In particular, Col3a1, Serpinh1, HMGCR, and FDPS may be future targets for reversing neural aging.

## Data Availability

The raw data has been uploaded to the SRA data bank in NCBI: https://dataview.ncbi.nlm.nih.gov/object/PRJNA980.

## LIST OF ABBREVIATIONS

SN: Sciatic Nerve
SD: Sprague Dawley
lncRNAs: Long non-coding RNAs
PPI: Protein-Protein Interaction
DEGs: Differentially Expressed Genes
PNs: Peripheral Nerves
DNA: Deoxyribonucleic Acid
NGS: Next-Generation RNA Sequencing
ncRNA: Non-Coding RNA
GO: Gene Ontology
