# Supplementary material for: Long Non-coding RNAs Influence Aging Process of Sciatic Nerves in SD Rats
Source: Comb Chem High Throughput Screen. 2023 Sep 7;27(14):2140–50. doi: 10.2174/1386207326666230907115800 (PMC11348477; doi:10.2174/1386207326666230907115800)
Supplement: Supplementary file 1 [file CCHTS-27-2140_SD1.pdf]

# Supplementary Material

# Long Non-coding RNAs Influence Aging Process of Sciatic Nerves in SD Rats

Rui Kuang[1,#], Yi Zhang[1,#], Guanggeng Wu[1,#], Zhaowei Zhu[1], Shuqia Xu[1], Xiangxia Liu[2,*], Yangbin Xu[1,*] and Yunxiang Luo[1,*]

[1]*Department of Plastic Surgery, The First Affiliated Hospital of Sun Yat-Sen University, No. 58 Zhongshan Road 2, Guangzhou 510080, China;* [2]*Department of Plastic Surgery, University of Tennessee Health Science Center, Memphis, TN, United States*

**Supplementary Table 1. The primer pair sequences in RT-qPCR.**

| Primers | Forward | Reverse |
|---|---|---|
| GAPDH | TTC CTA CCC CCA ATG TAT CCG | CAT GAG GTC CAC CAC CCT GTT |
| BTC | CCT TGT CCT GGG TCT TGT GATT | ATG CAG GAG GGA GTT TGT TCG |
| CDC20 | CAT TTG GAA CGT CTG CTC TGG | AGC TCA AAG CAG CGC CAG A |
| DLG2 | ATG ATC ATT CCT TAC CTC GGC TA | GTT GAC AAT TAT AGG AGC AGG GC |
| EGR2 | GCC AAG GCC GTA GAC AAA ATC | ATA TGG GAG ATC CAA GGG CCT |
| ACVR1C | TTG CGG CAG GAC TGA AGT GT | CTG TGG GAA GGT GCA GAG TGA T |
| JUN | CTT CTA CGA CGA TGC CCT CAA C | GGG TCG GTG TAG TGG TGA TGT G |
| MAL | CAG TGG CTT CTC CGT CTT CGT | TGT ACA TGA CCA TCA GGG AAG TG |
| MAP3K13 | ATC CAG TTC AGC AGG TCA GGC | TTC CTC AAA CGG CAC TTC CC |
| MBP | GCT TCT TTA GCG GTG ACA GGG | TGT GAG TCC TTG TAC ATG TGG CA |
| MPZ | GCT GCC CTG CTC TTC TCT TCT T | GGT TGA CCC TTG GCA TAG TGG A |
| NCMAP | CCG TCT TCT CGC TGA ACA TGA | CCT GGT CCT CAT TCT CCT GTT G |
| NGFR | CCT TGT GGC CTA TAT TGC TTT CA | AGG CAG TCT GCG TAT GGG TCT |
| PBK | GGA ATC AGT AAT TTC AAG ACG CC | AAT GGG ACA ACC CTC TCG GA |
| PDGFB | GAG CAT CGA GCC AAG ACA CCT | CCT TCT TGT CAT GGG TGT GCT T |
| PMP22 | TCG CGG TGC TAG TGT TGC TC | TGA AGC CAT TCG CTC ACA GAT |
| VEGF | AAT GAT GAA GCC CTG GAG TGC | TAA ACC GGG ATT TCT TGC GC |
| WWC1 | TGA GGA TGC TGG AGA AGA GGG T | GCA GAG AGA GCT GGG ATG TTC AT |

**Supplementary Table 2. GO enrichment analysis of up-regulated DElncRNAs.**

| Ontology | ID | Description | GeneRatio | | | P value | Fold Enrichment |
|---|---|---|---|---|---|---|---|
| BP | GO:0071219 | cellular response to molecule of bacterial origin | 2 | / | 29 | 1.31E-02 | 147.00 |
| BP | GO:0035988 | chondrocyte proliferation | 2 | / | 29 | 1.88E-02 | 101.77 |
| BP | GO:0016126 | sterol biosynthetic process | 2 | / | 29 | 2.31E-02 | 82.69 |
| BP | GO:0035987 | endodermal cell differentiation | 2 | / | 29 | 4.15E-02 | 45.62 |

| | | | | | | | |
|---|---|---|---|---|---|---|---|
| BP | GO:0007259 | JAK-STAT cascade | 2 | / | 29 | 4.43E-02 | 42.68 |
| BP | GO:1901216 | positive regulation of neuron death | 3 | / | 29 | 3.01E-03 | 35.44 |
| BP | GO:0051216 | cartilage development | 3 | / | 29 | 6.05E-03 | 24.81 |
| BP | GO:0046330 | positive regulation of JNK cascade | 3 | / | 29 | 9.31E-03 | 19.84 |
| BP | GO:0030198 | extracellular matrix organization | 4 | / | 29 | 2.24E-03 | 14.70 |
| BP | GO:0045766 | positive regulation of angiogenesis | 3 | / | 29 | 2.27E-02 | 12.40 |
| BP | GO:0009617 | response to bacterium | 3 | / | 29 | 3.87E-02 | 9.27 |
| BP | GO:0032355 | response to estradiol | 3 | / | 29 | 3.87E-02 | 9.27 |
| BP | GO:0008284 | positive regulation of cell proliferation | 5 | / | 29 | 1.06E-02 | 5.52 |
| CC | GO:0044297 | cell body | 3 | / | 28 | 1.25E-02 | 17.00 |
| CC | GO:0031012 | extracellular matrix | 6 | / | 28 | 3.56E-05 | 15.02 |
| CC | GO:0045121 | membrane raft | 4 | / | 28 | 8.37E-03 | 9.14 |
| CC | GO:0009986 | cell surface | 5 | / | 28 | 1.39E-02 | 5.09 |
| CC | GO:0005576 | extracellular region | 5 | / | 28 | 2.86E-02 | 4.09 |
| MF | GO:0005127 | ciliary neurotrophic factor receptor binding | 2 | / | 27 | 1.47E-02 | 129.93 |
| MF | GO:0070402 | NADPH binding | 2 | / | 27 | 2.78E-02 | 68.39 |

**Supplementary Table 3. GO enrichment analysis of down-regulated DElncRNAs.**

| Ontology | ID | Description | GeneRatio | | | P value | Fold Enrichment |
|---|---|---|---|---|---|---|---|
| BP | GO:0060841 | venous blood vessel development | 3 | / | 873 | 2.74E-02 | 10.99 |
| BP | GO:0010867 | positive regulation of triglyceride biosynthetic process | 7 | / | 873 | 1.01E-04 | 8.55 |
| BP | GO:0045542 | positive regulation of cholesterol biosynthetic process | 4 | / | 873 | 1.17E-02 | 7.99 |
| BP | GO:0022011 | myelination in peripheral nervous system | 8 | / | 873 | 5.06E-05 | 7.64 |
| BP | GO:0090181 | regulation of cholesterol metabolic process | 4 | / | 873 | 3.37E-02 | 5.49 |
| BP | GO:1900271 | regulation of long-term synaptic potentiation | 4 | / | 873 | 3.37E-02 | 5.49 |
| BP | GO:0030199 | collagen fibril organization | 10 | / | 873 | 6.71E-04 | 4.07 |
| BP | GO:0001570 | vasculogenesis | 9 | / | 873 | 1.89E-02 | 2.67 |
| BP | GO:0001568 | blood vessel development | 9 | / | 873 | 2.03E-02 | 2.64 |
| BP | GO:0008203 | cholesterol metabolic process | 9 | / | 873 | 2.18E-02 | 2.60 |
| BP | GO:0010977 | negative regulation of neuron projection development | 9 | / | 873 | 4.66E-02 | 2.25 |
| BP | GO:0006629 | lipid metabolic process | 11 | / | 873 | 2.97E-02 | 2.18 |
| BP | GO:0030198 | extracellular matrix organization | 17 | / | 873 | 8.13E-03 | 2.08 |
| BP | GO:0001525 | angiogenesis | 19 | / | 873 | 1.13E-02 | 1.91 |
| BP | GO:0007399 | nervous system development | 19 | / | 873 | 2.34E-02 | 1.76 |
| CC | GO:0005588 | collagen type V trimer | 3 | / | 901 | 1.16E-02 | 16.51 |
| CC | GO:0031012 | extracellular matrix | 29 | / | 901 | 9.60E-05 | 2.26 |
| CC | GO:0045202 | synapse | 53 | / | 901 | 1.54E-05 | 1.88 |
| MF | GO:0005201 | extracellular matrix structural constituent | 10 | / | 804 | 3.34E-03 | 3.26 |
| MF | GO:0005518 | collagen binding | 10 | / | 804 | 4.50E-03 | 3.12 |

**Supplementary Table 4. KEGG enrichment analysis of up-regulated DElncRNAs.**

| ID | Description | GeneRatio | | | P value | Fold Enrichment |
|---|---|---|---|---|---|---|
| rno04922 | Glucagon signaling pathway | 5 | / | 99 | 2.75E-02 | 4.32 |
| rno04921 | Oxytocin signaling pathway | 6 | / | 99 | 2.80E-02 | 3.48 |
| rno04621 | NOD-like receptor signaling pathway | 6 | / | 99 | 4.54E-02 | 3.05 |
| rno05417 | Lipid and atherosclerosis | 7 | / | 99 | 2.75E-02 | 3.01 |
| rno01100 | Metabolic pathways | 26 | / | 99 | 3.72E-02 | 1.47 |

**Supplementary Table 5. KEGG enrichment analysis of dowm-regulated DElncRNAs**

| ID | Description | GeneRatio | | | P value | Fold Enrichment |
|---|---|---|---|---|---|---|
| rno00900 | Terpenoid backbone biosynthesis | 6 | / | 438 | 6.55E-03 | 4.88 |
| rno04927 | Cortisol synthesis and secretion | 11 | / | 438 | 1.65E-03 | 3.29 |
| rno00513 | Various types of N-glycan biosynthesis | 6 | / | 438 | 4.46E-02 | 3.05 |
| rno04974 | Protein digestion and absorption | 15 | / | 438 | 4.79E-04 | 2.96 |
| rno04933 | AGE-RAGE signaling pathway in diabetic complications | 14 | / | 438 | 1.27E-03 | 2.82 |

**Supplementary Table 6. Table of abbreviations.**

| Full Name | Abbreviations |
|---|---|
| biological process | BP |
| cellular component | CC |
| collagen | Col |
| competing endogenous RNA | ceRNA |
| deoxyribonucleic acid | DNA |
| differentially expressed genes | DEGs |
| differentially expressed lncRNA | DElncRNA |
| differentially expressed mRNA | DEmRNA |
| extracellular matrix | ECM |
| gene ontology | GO |
| Institutional Animal Care and Use Committee | IACUC |
| long non-coding RNA | lncRNA |
| messenger ribonucleic acid | Mrna |
| messenger RNA | mRNA |
| next-generation RNA sequencing | NGS |
| non-coding RNA | ncRNA |
| Pearson correlation coefficient | PCC |
| peripheral nerves | PNs |
| Polymerase Chain Reaction | PCR |
| protein-protein interaction | PPI |
| real-time-polymerase chain reaction | qRT-PCR |
| schwann cell | SC |
| sciatic nerve | SN |
| Sprague Dawley | SD |
